# Maternal Milk Orchestrates the Development of Infant Gut Microbiota: Implications for Health and Future Research Directions

**DOI:** 10.34133/research.0558

**Published:** 2025-02-19

**Authors:** Ran Wang, Siyuan Sun, Qi Zhang, Guanglei Wu, Fazheng Ren, Juan Chen

**Affiliations:** ^1^ Key Laboratory of Precision Nutrition and Food Quality, Department of Nutrition and Health, China Agricultural University, Beijing 100190, China.; ^2^ College of Food Science and Nutritional Engineering, Key Laboratory of Functional Dairy, Co-constructed by Ministry of Education and Beijing Government, China Agricultural University, Beijing 100083, China.

## Abstract

Human breast milk serves as a vital source of nutrition for infants, and it also plays a critical role in shaping the infant gut microbiota and establishing intestinal homeostasis. This process substantially impacts immune function, neurodevelopment, and overall health. The noninvasive nature of breast milk collection makes it an ethical and accessible area for research, positioning it as a key focus for future studies. These future directions include the identification of novel bacteria combination, the establishment of comprehensive databases on infant microbiota, and the use of computational models to predict interactions between breast milk components and the gut microbiome. Additionally, the creation of diverse biological models and the establishment of infant stool banks will further enhance understanding of host–microbiome interactions and support disease prevention strategies.

## Introduction

Human breast milk (HBM) is a complex, dynamic fluid that plays a pivotal role in infant nutrition, immune system development, and overall health [1]. It contains a wide range of bioactive components that support both immediate nutritional needs and long-term health benefits. The primary macronutrients in HBM include carbohydrates, predominantly in the form of lactose, which provides energy and aids in the development of the infant’s gut microbiota. Proteins, including casein and whey proteins, are vital for growth and immune protection, as they contain immunoglobulins, enzymes, and growth factors that enhance infant immunity and development. Fats provide essential fatty acids like docosahexaenoic acid (DHA) and arachidonic acid (ARA), crucial for brain and retinal development [2]. Besides essential vitamins and minerals as micronutrients, a unique feature of HBM is the presence of human milk oligosaccharides (HMOs), which are indigestible by the infant but serve as prebiotics, promoting the growth of beneficial gut bacteria and contributing to immune defense by preventing pathogen colonization [3,4]. HBM also contains immunomodulating components, including antibodies, cytokines, and leukocytes, helps protect the infant from infections, and supports the maturation of the immune system [5]. Breast milk also contains hormones and growth factors like insulin, leptin, and epidermal growth factor (EGF), which play roles in metabolic regulation and tissue development [6]. The composition of breast milk is not static [7]; it changes over time to meet the evolving needs of the growing infant, making it a unique and tailored source of nourishment that adapts to developmental stages, from colostrum to mature milk.

The infant gut microbiota is characterized by its simplicity, dynamic nature, and significant evolution during the first few years of life. Initially, at birth, the normal infant’s gut is sterile [8]. During the vaginal section, the bacteria in the mother’s vagina will spread to the skin and the gut intestinal tract of newborns [9]. The microorganisms in the breast milk are then transported into the babies’ gut by breastfeeding [10]. The first microbes to colonize the infant gut are generally facultative anaerobes such as *Enterococcus*, *Streptococcus*, and *Staphylococcus* [11]. In vaginally delivered infants, microbes from the mother’s vaginal and fecal flora dominate, whereas cesarean-delivered infants are initially colonized by skin-associated bacteria like *Staphylococcus* and *Corynebacterium* [12]. As the babies constantly consume HBM rich in HMOs, *Bifidobacterium* and *Lactobacillus*, which thrive on HMOs, are dominant in the infant’s microbial composition. As solid foods are introduced around 6 months, the diversity of the gut microbiota expands. Bacterial taxa associated with plant fiber digestion, such as the phylum of Bacteroidetes and Firmicutes, begin to increase. The gut microbiota undergoes significant shifts during this period, transitioning from a milk-based microbial profile to one that can metabolize more complex carbohydrates and other nutrients found in solid foods [13].

## HBM Nourishes the Infants in Multiple Ways

**Fig. 1.**
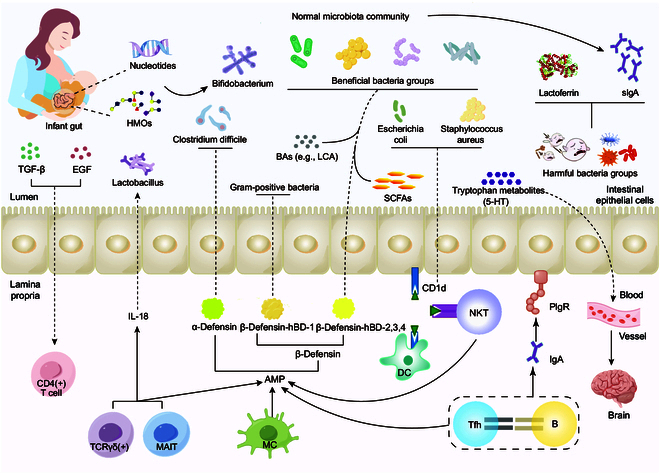
HBM plays a crucial role in the overall infant gut environment. The HBM-derived nucleotides help maintain the integrity of gut barrier. LF and sIgA in the HBM can bind the harmful microorganisms and clear them subsequently. HMOs promote the growth of beneficial bacteria like *Bifidobacterium*, which in turn increase the level of functional metabolites such as SCFAs, bile acids (BAs), and 5-HT. Those metabolites contribute to the intestinal homeostasis, the precise response of immune system, and the development of neural system. PIgR, polymeric immunoglobulin receptor; AMP, antimicrobial peptide; TCRγδ(+), T cell receptor γδ-positive cell; MAIT, mucosal-associated invariant T cell; MC, macrophage cell; Tfh, follicular helper T cell; NKT, natural killer T cell; IL-18, interleukin-18; CD1d, cluster of differentiation 1d.

The substances in HBM actively shape the composition of the infant gut microbes, which has a lasting effect on intestinal homeostasis, immune function, and neurological development (Fig. 1).

### The maintenance of intestinal homeostasis

HBM plays a critical role in regulating infant intestinal homeostasis through multiple mechanisms that support both the development of the gut microbiota and the maturation of the intestinal barrier and immune system. Key components of breast milk involved in this regulation include HMOs, nucleotides, and lactoferrin (LF). HMOs are complex sugars that are indigestible by the infant but can selectively feed beneficial gut bacteria like *Bifidobacterium*. For example, *Bifidobacterium longum* subsp. *infantis* expresses fucosyllactose transporters that enable this species to utilize 2′-fucosyllactose (2′-FL), one of the most abundant HMOs [14]. Some coexisting species can encode extracellular fucosidases to hydrolyze 2′-FL, contributing to the growth of *Bifidobacterium breve* [15]. These beneficial microbes help maintain a healthy gut environment by producing short-chain fatty acids (SCFAs) that lower gut pH, inhibiting pathogen growth and absorbed by intestinal epithelial cells as an energy source [16]. Therefore, the intestinal barrier integrity can be achieved. Aside from HMOs, nucleotides in HBM can also reshape the composition of an infant’s gut microbiota. To act as essential growth factors, nucleotides enhance the abundance of beneficial microbes like *Bifidobacterium* [17]. Nucleotides also support the structural and functional development of the infant’s intestinal lining, building up the gut barrier and improving nutrient uptake [18]. LF, which is one component of HBM, serves as an antimicrobial substance by disrupting the biofilms of enteropathogenic bacteria [19]. By promoting the growth of specific microbes, HBM facilitates the development of a gut microbiome that is favorable to the infant’s intestinal tract health.

### The strengthening of immune system

When the infants are born, they bear immune deficiencies like immature phagocyte function and inadequate immunocyte responses. Fortunately, HBM contains higher amounts of macrophages, immunoglobulins, particularly secretory immunoglobulin A (sIgA), which coat the mucosal lining of the infant’s gut [20]. *Streptococcus agalactiae*, a major etiological agent of neonatal sepsis, can be effectively inhibited by maternal sIgA, since sIgA prevents pathogenic colonization of the intestine and subsequent translocation to other sites [21]. LF, another key protein, transports ions in the intestine. It exerts antibacterial effects against a wide range of microorganisms and neutralizes iron-mediated free radicals [22], thereby contributing to lower levels of inflammation in the infants. Breast milk contains live bacteria, mainly lactic acid bacteria, that maintain gut health by directly inhibiting pathogen growth along with antimicrobial peptides, like α-defensin and β-defensin [23,24]. Cytokines such as transforming growth factor-β (TGF-β) in breast milk promote the development of the infant’s immune system while maintaining a balanced immune response. Mediated by CD4^+^ T lymphocytes, milk-borne TGF-β can reduce the risk of overactive immune reactions, such as allergic airway disease [25]. TGF-β, together with EGF and fibroblast growth factor, elicits the maturation of lymphocytes in mesenteric lymph nodes [26], establishing a robust intestinal barrier that prevents pathogens from entering the bloodstream.

### The development of neural system

Studies in both humans and animal models suggest that early disruptions in the gut microbiome can influence neurodevelopmental outcomes, potentially affecting cognitive functions like learning and memory, as well as emotional regulation [27]. Moreover, gut dysbiosis has been increasingly linked to the development of neurodevelopmental and neuropsychiatric disorders such as autism spectrum disorder (ASD), anxiety, and other neural diseases through the gut–brain axis [28]. This axis involves bidirectional communication between the gastrointestinal tract and the central nervous system (CNS), mediated by neural, endocrine, immune, and metabolic pathways [29]. Studies have demonstrated the significant effect of HBM on the infant microbiome, which impacts brain and behavior development [30–32]. By utilizing substances in HBM, the infant’s gut microbiota can produce beneficial compounds, such as SCFAs and bile acids, which stimulate the release of 5-hydroxytryptamine (5-HT) [33], enhancing synaptogenesis and neuronal progenitor cell proliferation and improving the overall cognitive function [34].

## Insights into Future Infant Microbiome Studies

### Expand the application of gnotobiotic mouse model

**Fig. 2.**
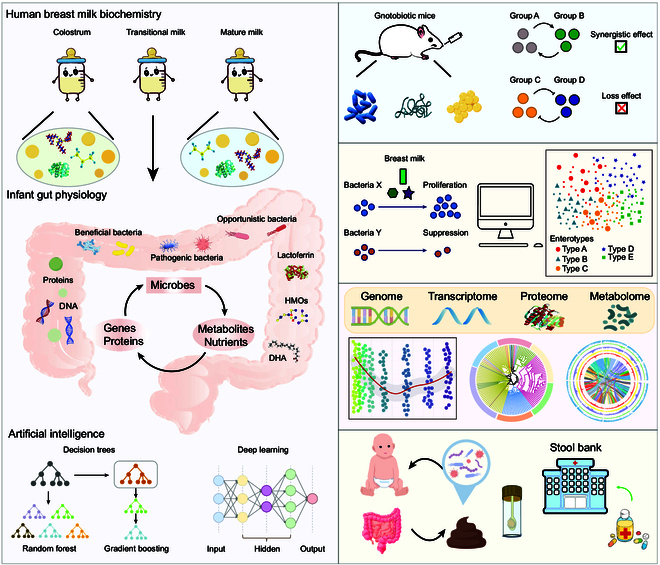
Future methods exploring the interaction between HBM and infant gut microbiota. (1) Using the GN model to investigate the mutual influence of different groups of bacteria. (2) Establishing a database to feature the components of HBM in different stages and to identify which bacteria can utilize HBM substances and have a positive effect on the host. (3) Developing new computational models with the use of AI to describe the dynamics of colonization of gut microbes. (4) Building up infant stool bank to restore the gut microbiota of healthy infants.

Future research should extensively utilize gnotobiotic mouse (GN) models to investigate the impact of specific microorganisms on infant gut microbiota. With the advent of GN models—whereby germ-free mice are administered one or several specific strains—researchers have primarily focused on mature adult gut environments characterized by complex microbial communities [35–37], rather than on developing intestinal systems. However, given the limited diversity and significantly lower abundance of microbial species in neonatal guts compared to adults, this biological model is better suited to simulate the dynamics of gut microbiota in infants. By integrating this model with infant mouse models, researchers can identify beneficial microorganisms that promote the healthy development of the infant gut or metabolize HBM, like HMOs, with high efficiency. For instance, researchers can assess the collective impact of specific bacterial species on the host by administering a consortium of selected bacteria using the aforementioned model. This approach allows for the evaluation of synergistic effects among the bacteria, providing a more comprehensive understanding of how these microbial communities interact with the host’s physiology and contribute to overall health. These studies can assess whether the coexistence of multiple bacterial species produces beneficial effects, thereby shedding light on the underlying mechanisms of microbiome–host interactions (Fig. 2).

### AI prediction of the interaction between human milk substances and infant gut microbiota

Current research employs computer-simulated molecular docking to assess the binding of certain HMOs to their ligands in the host gastrointestinal tract [38]. Future studies should leverage computer technology and artificial intelligence (AI) to establish connections between infant gut microbiota and breast milk: (a) utilizing AI methods to develop models of cross-feeding interactions between HMOs and gut microbiota; (b) examining the effects of the content and ratios of biomarkers (e.g., inflammatory factors) in breast milk on gut microbiota. In the present, infant formulae typically incorporate only a single strain or type of probiotic. However, gut microbiota generally engage in physiological metabolism through cross-feeding mechanisms [39,40]. Additionally, the colonization of a single bacterial strain makes the gut more susceptible to foreign microbial invasion, whereas probiotic consortia can enhance the overall disease resistance and antibacterial capacity of the host’s gut. Therefore, future formulations of infant formula should incorporate a wide variety of probiotics beyond just *Bifidobacterium*, *Lactobacillus*, *Akkermansia*, etc.

Given the inherent variability in breast milk composition throughout lactation, fluctuations in the concentration of specific components, such as HMOs, can influence the growth trajectories of bacteria colonizing the neonatal gut. Computational modeling has emerged as a valuable tool for analyzing the dose-dependent interactions between these bacteria and their nutrient sources, allowing for a more precise understanding of how variations in milk composition affect microbial colonization dynamics. Furthermore, a database of common constituents in breast milk could be established, with certain components identified as biomarkers for adverse effects on infants. AI and relevant algorithms could then be employed to predict whether specific constituents in breast milk might have negative impacts on infant health.

### New methodology to characterize the patterns of migration of gut microbiota in early life

A combined approach utilizing multi-omics and AI can be employed to model the migration and dynamic transformation of gut microbiota in newborns during early development. Previous studies tend to utilize genomics to explore the pattern of the infant’s microbiome [41]. By integrating various layers of biological data—such as genomics, transcriptomics, proteomics, and metabolomics—advanced method should offer a comprehensive view of how microbial communities shift and evolve in the infant gut over time. AI, with its capacity for complex data analysis and pattern recognition, will be crucial in identifying intricate interactions between microbiota and host factors.

The establishment of a robust database that records these microbial shifts in newborns’ gut ecosystems will serve as a valuable resource for researchers. Such a database could be used to track how the introduction of specific microbial species or groups of microorganisms at various stages of early life affects the overall microbiota composition. Research can adopt an ecological approach by utilizing an invasive model to simulate the changes in the infant gut microbiota following the introduction of a specific microorganism, whether it be a beneficial probiotic or a pathogenic bacterium. This method allows for the analysis of how newly introduced microbes interact with the existing gut ecosystem, providing valuable insights into the dynamics of microbial colonization and its implications for infant health. For instance, the impact of introducing a particular strain of *Bifidobacterium* during the first weeks or months of life could be analyzed for its effects on long-term microbiome stability, immune development, or metabolic health. These studies could provide valuable evidence for preventing or mitigating future health conditions, such as allergies, obesity, or autoimmune diseases.

### Is it possible to establish stool banking for infants?

The stool banking necessitates long-term stool storage for individuals who have the potential to gut dysbiosis. Recently, some studies have employed the method of autologous fecal microbiota transplantation (aFMT), where an individual’s microbiota is preserved in advance and later reintroduced following antibiotic treatment to restore the gut microbiota [42]. While FMT has been widely studied in adult populations [43,44], its application in infants is still limited, especially when it comes to aFMT. In fact, it is reasonable to deduce that infants could also benefit from aFMT to alleviate the adverse symptoms caused by gastrointestinal disorders. Given the relatively simple composition of the gut microbiota in infants, it is more feasible to preserve the majority of the bacterial consortium. In the future, the establishment of an infant microbiota reservoir should be pursued, paving the way for aFMT in infants as a potential strategy for treating some highly prevalent diseases like necrotizing enterocolitis (NEC) and inflammatory bowel disease (IBD) [45]. This concept could represent a promising direction for industry development aimed at improving children’s overall health and immunity.

## Concluding Remarks

HBM is an indispensable source of nutrition for infants, playing a crucial role in shaping the gut microbiota and establishing intestinal homeostasis, which in turn significantly influences the infant’s overall development. Due to its noninvasive collection process, the study of breast milk does not harm the subjects, making it a highly accessible and ethical area of research. Future research is likely to focus on identifying new beneficial bacteria groups, creating a comprehensive database of infant gut microbiota, and utilizing computational modeling to predict interactions between HBM components and the infant microbiome. By identifying the optimal probiotic combinations, the development of innovative infant formula regulating gut microbiome can be significantly boosted. Furthermore, the development of diverse biological models could enhance the understanding of host–microbiome interactions. From a therapeutic perspective, the establishment of infant stool banks could serve as a critical resource for maintaining gut health of babies and supporting disease prevention strategies.

## References

[1] Perrella S, Gridneva Z, Lai CT, Stinson L, George A, Bilston-John S, Geddes D. Human milk composition promotes optimal infant growth, development and health. Semin Perinatol. 2021;45(2): Article 151380. 33431112 10.1016/j.semperi.2020.151380

[2] Liu W, Zeng T, Mueed A, Zhang B, Wei T, Deng Z, Xi Q. Dynamic changes at high-protein dietary pattern of major fatty acids in healthy lactating women: A systematic review and meta-analysis. Nutrition. 2024;121: Article 112362. 38354680 10.1016/j.nut.2024.112362

[3] Sakarya E, Sanlier NT, Sanlier N. The relationship between human milk, a functional nutrient, and microbiota. Crit Rev Food Sci Nutr. 2023;63:4842–4854. 34872407 10.1080/10408398.2021.2008301

[4] Le Doare K, Holder B, Bassett A, Pannaraj PS. Mother’s milk: A purposeful contribution to the development of the infant microbiota and immunity. Front Immunol. 2018;9:361. 29599768 10.3389/fimmu.2018.00361 PMC5863526

[5] Chen Y, Wen Y, Zhao R, Zhu Y, Chen Z, Zhao C, Mu W. Human milk oligosaccharides in preventing food allergy: A review through gut microbiota and immune regulation. Int J Biol Macromol. 2024;278(Pt 2): Article 134868. 39163965 10.1016/j.ijbiomac.2024.134868

[6] Orofiamma LA, Vural D, Antonescu CN. Control of cell metabolism by the epidermal growth factor receptor. Biochim Biophys Acta Mol Cell Res. 2022;1869: Article 119359. 36089077 10.1016/j.bbamcr.2022.119359

[7] Ballard O, Morrow AL. Human milk composition: Nutrients and bioactive factors. Pediatr Clin N Am. 2013;60(12):49–74. 10.1016/j.pcl.2012.10.002 PMC3586783 23178060

[8] Perez-Munoz ME, Arrieta MC, Ramer-Tait AE, Walter J. A critical assessment of the “sterile womb” and “in utero colonization” hypotheses: Implications for research on the pioneer infant microbiome. Microbiome. 2017;5(1):48. 28454555 10.1186/s40168-017-0268-4 PMC5410102

[9] Zhu B, Edwards DJ, Spaine KM, Edupuganti L, Matveyev A, Serrano MG, Buck GA. The association of maternal factors with the neonatal microbiota and health. Nat Commun. 2024;15(1):5260. 38898021 10.1038/s41467-024-49160-w PMC11187136

[10] Laursen MF, Pekmez CT, Larsson MW, Lind MV, Yonemitsu C, Larnkjaer A, Molgaard C, Bode L, Dragsted LO, Michaelsen KF, et al. Maternal milk microbiota and oligosaccharides contribute to the infant gut microbiota assembly. ISME Commun. 2021;1(2):21. 36737495 10.1038/s43705-021-00021-3 PMC9723702

[11] Boudry G, Charton E, Le Huerou-Luron I, Ferret-Bernard S, Le Gall S, Even S, Blat S. The relationship between breast milk components and the infant gut microbiota. Front Nutr. 2021;8: Article 629740. 33829032 10.3389/fnut.2021.629740 PMC8019723

[12] Shin H, Pei Z, Martinez KA II, Rivera-Vinas JI, Mendez K, Cavallin H, Dominguez-Bello MG, Rivera-Vinas JI, Mendez K, Cavallin H, et al. The first microbial environment of infants born by C-section: The operating room microbes. Microbiome. 2015;3:59. 26620712 10.1186/s40168-015-0126-1 PMC4665759

[13] Yatsunenko T, Rey FE, Manary MJ, Trehan I, Dominguez-Bello MG, Contreras M, Magris M, Hidalgo G, Baldassano RN, Anokhin AP, et al. Human gut microbiome viewed across age and geography. Nature. 2012;486(7402):222–227. 22699611 10.1038/nature11053 PMC3376388

[14] Sakanaka M, Hansen ME, Gotoh A, Katoh T, Yoshida K, Odamaki T, Yachi H, Sugiyama Y, Kurihara S, Hirose J, et al. Evolutionary adaptation in fucosyllactose uptake systems supports bifidobacteria-infant symbiosis. Sci Adv. 2019;5(8): Article eaaw7696. 31489370 10.1126/sciadv.aaw7696 PMC6713505

[15] Lou YC, Rubin BE, Schoelmerich MC, DiMarco KS, Borges AL, Rovinsky R, Song L, Doudna JA, Banfield JF. Infant microbiome cultivation and metagenomic analysis reveal Bifidobacterium 2′-fucosyllactose utilization can be facilitated by coexisting species. Nat Commun. 2023;14(1):7417. 37973815 10.1038/s41467-023-43279-y PMC10654741

[16] Zhang Q, Li G, Zhao W, Wang X, He J, Zhou L, Zhang X, An P, Liu Y, Zhang C, et al. Efficacy of Bifidobacterium animalis subsp. lactis BL-99 in the treatment of functional dyspepsia: A randomized placebo-controlled clinical trial. Nat Commun. 2024;15(1):227. 38172093 10.1038/s41467-023-44292-x PMC10764899

[17] Qu Z, Zhang B, Lin G, Guo M, Tian P, Wang L, Chen W, Zhang H, Wang G. Dietary nucleotides drive changes in infant fecal microbiota in vitro and gut microbiota-gut-brain development in neonatal rats: A potential “nitrogen source” for early microbiota growth. Food Chem. 2024;463(Pt 3): Article 141333. 39340921 10.1016/j.foodchem.2024.141333

[18] Xu M, Ma Y, Xu L, Xu Y, Li Y. Developmental effects of dietary nucleotides in second-generation weaned rats. J Med Food. 2013;16(12):1146–1152. 24328704 10.1089/jmf.2013.2790 PMC3868252

[19] Li B, Zhang B, Zhang F, Liu X, Zhang Y, Peng W, Teng D, Mao R, Yang N, Hao Y, et al. Interaction between dietary lactoferrin and gut microbiota in host health. J Agric Food Chem. 2024;72(14):7596–7606. 38557058 10.1021/acs.jafc.3c09050

[20] Jakaitis BM, Denning PW. Human breast milk and the gastrointestinal innate immune system. Clin Perinatol. 2014;41(2):423–435. 24873841 10.1016/j.clp.2014.02.011 PMC4414019

[21] Greenfield KG, Harlow OS, Witt LT, Dziekan EM, Tamar CR, Meier J, Brumbaugh JE, Levy ER, Knoop KA. Neonatal intestinal colonization of Streptococcus agalactiae and the multiple modes of protection limiting translocation. Gut Microbes. 2024;16(1):2379862. 39042143 10.1080/19490976.2024.2379862 PMC11268251

[22] Cao X, Ren Y, Lu Q, Wang K, Wu Y, Wang Y, Zhang Y, Cui XS, Yang Z, Chen Z. Lactoferrin: A glycoprotein that plays an active role in human health. Front Nutr. 2022;9:1018336. 36712548 10.3389/fnut.2022.1018336 PMC9875800

[23] Yu J, Li W, Xu R, Liu X, Gao G, Kwok LY, Chen Y, Sun Z, Liu W, Zhang H. Probio-M9, a breast milk-originated probiotic, alleviates mastitis and enhances antibiotic efficacy: Insights into the gut-mammary axis. iMeta. 2024;3(4): Article e224. 39135694 10.1002/imt2.224 PMC11316926

[24] Qiao N, Du G, Zhong X, Sun X. Recombinant lactic acid bacteria as promising vectors for mucosal vaccination. Exploration (Beijing). 2021;1:20210026. 37323212 10.1002/EXP.20210026 PMC10191043

[25] Oddy WH, Rosales F. A systematic review of the importance of milk TGF-beta on immunological outcomes in the infant and young child. Pediatr Allergy Immunol. 2010;21(1 Pt 1):47–59. 19594862 10.1111/j.1399-3038.2009.00913.x

[26] Torres-Castro P, Abril-Gil M, Rodriguez-Lagunas MJ, Castell M, Perez-Cano FJ, Franch A. TGF-beta2, EGF, and FGF21 growth factors present in breast milk promote mesenteric lymph node lymphocytes maturation in suckling rats. Nutrients. 2018;10(9):1171. 30150532 10.3390/nu10091171 PMC6163676

[27] Lu J, Claud EC. Connection between gut microbiome and brain development in preterm infants. Dev Psychobiol. 2019;61(5):739–751. 30460694 10.1002/dev.21806 PMC6728148

[28] O’Mahony SM, Stilling RM, Dinan TG, Cryan JF. The microbiome and childhood diseases: Focus on brain-gut axis. Birth Defects Res C Embryo Today. 2015;105(4):296–313. 26706413 10.1002/bdrc.21118

[29] Yang L, Hung LY, Zhu Y, Ding S, Margolis KG, Leong KW. Material engineering in gut microbiome and human health. Research. 2022;2022:9804014. 35958108 10.34133/2022/9804014 PMC9343081

[30] Sindi AS, Geddes DT, Wlodek ME, Muhlhausler BS, Payne MS, Stinson LF. Can we modulate the breastfed infant gut microbiota through maternal diet? FEMS Microbiol Rev. 2021;45(5): Article fuab011. 33571360 10.1093/femsre/fuab011

[31] Lundgren SN, Madan JC, Emond JA, Morrison HG, Christensen BC, Karagas MR, Hoen AG. Maternal diet during pregnancy is related with the infant stool microbiome in a delivery mode-dependent manner. Microbiome. 2018;6:109. 29973274 10.1186/s40168-018-0490-8 PMC6033232

[32] Isaacs EB, Fischl BR, Quinn BT, Chong WK, Gadian DG, Lucas A. Impact of breast milk on intelligence quotient, brain size, and white matter development. Pediatr Res. 2010;67(4):357–362. 20035247 10.1203/PDR.0b013e3181d026da PMC2939272

[33] Yano JM, Yu K, Donaldson GP, Shastri GG, Ann P, Ma L, Nagler CR, Ismagilov RF, Mazmanian SK, Hsiao EY. Indigenous bacteria from the gut microbiota regulate host serotonin biosynthesis. Cell. 2015;161(2):264–276. 25860609 10.1016/j.cell.2015.02.047 PMC4393509

[34] Brummelte S, Mc Glanaghy E, Bonnin A, Oberlander TF. Developmental changes in serotonin signaling: Implications for early brain function, behavior and adaptation. Neuroscience. 2017;342:212–231. 26905950 10.1016/j.neuroscience.2016.02.037 PMC5310545

[35] Culp EJ, Nelson NT, Verdegaal AA, Goodman AL. Microbial transformation of dietary xenobiotics shapes gut microbiome composition. Cell. 2024;187(22):6327–6345.e20. 39321800 10.1016/j.cell.2024.08.038 PMC11531382

[36] Grant ET, Parrish A, Boudaud M, Hunewald O, Hirayama A, Ollert M, Fukuda S, Desai MS. Dietary fibers boost gut microbiota-produced B vitamin pool and alter host immune landscape. Microbiome. 2024;12(1):179. 39307855 10.1186/s40168-024-01898-7 PMC11418204

[37] Savage HP, Bays DJ, Tiffany CR, Gonzalez MAF, Bejarano EJ, Carvalho TP, Luo Z, Masson HLP, Nguyen H, Santos RL, et al. Epithelial hypoxia maintains colonization resistance against Candida albicans. Cell Host Microbe. 2024;32(7):1103–1113.e6. 38838675 10.1016/j.chom.2024.05.008 PMC11239274

[38] Endo S, Sugita T, Kamai S, Nakamura K, Yamazaki F, Sampei S, Snarskis G, Valanciute A, Kazemi M, Rokaitis I, et al. Selective microbial production of lacto-N-fucopentaose I in Escherichia coli using engineered alpha-1,2-fucosyltransferases. Metab Eng. 2024;82:1–11. 38145749 10.1016/j.ymben.2023.12.009

[39] Sinha AK, Laursen MF, Brinck JE, Rybtke ML, Hjorne AP, Prochazkova N, Pedersen M, Roager HM, Licht TR. Dietary fibre directs microbial tryptophan metabolism via metabolic interactions in the gut microbiota. Nat Microbiol. 2024;9(8):1964–1978. 38918470 10.1038/s41564-024-01737-3 PMC11306097

[40] Khan MT, Dwibedi C, Sundh D, Pradhan M, Kraft JD, Caesar R, Tremaroli V, Lorentzon M, Backhed F. Synergy and oxygen adaptation for development of next-generation probiotics. Nature. 2023;620(7973):381–385. 37532933 10.1038/s41586-023-06378-w PMC10412450

[41] Johnson KE, Heisel T, Allert M, Furst A, Yerabandi N, Knights D, Jacobs KM, Lock EF, Bode L, Fields DA, et al. Human milk variation is shaped by maternal genetics and impacts the infant gut microbiome. Cell Genom. 2024;4: Article 100638. 39265573 10.1016/j.xgen.2024.100638 PMC11602576

[42] Suez J, Zmora N, Zilberman-Schapira G, Mor U, Dori-Bachash M, Bashiardes S, Zur M, Regev-Lehavi D, Ben-Zeev Brik R, Federici S, et al. Post-antibiotic gut mucosal microbiome reconstitution is impaired by probiotics and improved by autologous FMT. Cell. 2018;174(6):1406–1423.e16. 30193113 10.1016/j.cell.2018.08.047

[43] Scheperjans F, Levo R, Bosch B, Laaperi M, Pereira PAB, Smolander OP, Aho VTE, Vetkas N, Toivio L, Kainulainen V, et al. Fecal microbiota transplantation for treatment of Parkinson disease: A randomized clinical trial. JAMA Neurol. 2024;81(9):925–938. 39073834 10.1001/jamaneurol.2024.2305 PMC11287445

[44] Zuppi M, Vatanen T, Wilson BC, Golovina E, Portlock T, Cutfield WS, Vickers MH, O’Sullivan JM. Fecal microbiota transplantation alters gut phage communities in a clinical trial for obesity. Microbiome. 2024;12(1):122. 38970126 10.1186/s40168-024-01833-w PMC11227244

[45] Liu Y, Jiao C, Zhang T, Li X, Li P, Lu M, Ye Z, Du Y, Du R, Zhang W, et al. Early-life gut microbiota governs susceptibility to colitis via microbial-derived ether lipids. Research. 2023;6:0037. 37040489 10.34133/research.0037 PMC10076029

